# Changes in cervical alignment of Zero-profile device versus conventional cage-plate construct after anterior cervical discectomy and fusion: a meta-analysis

**DOI:** 10.1186/s13018-022-03400-1

**Published:** 2022-11-24

**Authors:** Ziwen Liu, Yuming Yang, Jie Lan, Hanpeng Xu, Zepei Zhang, Jun Miao

**Affiliations:** Department of Spine Surgery, Tianjin Hospital, Jiefangnanlu 406, Hexi District, Tianjin, China

**Keywords:** Zero-profile, Anterior cervical discectomy and fusion, Cervical spondylotic myelopathy, Cervical alignment, Dysphagia

## Abstract

**Background:**

Anterior cervical diskectomy and fusion (ACDF) has been widely accepted as a gold standard for patients with cervical spondylotic myelopathy (CSM). However, there was insufficient evidence to compare the changes in the cervical alignment with different fusion devices in a long follow-up period. This meta-analysis was performed to compare the radiologic outcomes and loss of correction (LOC) in cervical alignment of Zero-profile (ZP) device versus cage-plate (CP) construct for the treatment of CSM.

**Methods:**

Retrospective and prospective studies directly comparing the outcomes between the ZP device and CP construct in ACDF were included. Data extraction was conducted and study quality was assessed independently. A meta-analysis was carried out by using fixed effects and random effects models to calculate the odds ratio and mean difference in the ZP group and the CP group.

**Results:**

Fourteen trials with a total of 1067 participants were identified. ZP group had a lower rate of postoperative dysphagia at the 2- or 3-month and 6-month follow-up than CP group, and ZP group was associated with a decreased ASD rate at the last follow-up when compared with the CP group. The pooled data of radiologic outcomes revealed that there was no significant difference in postoperative and last follow-up IDH. However, postoperative and last follow-up cervical Cobb angle was significantly smaller in the ZP group when compared with the CP group. In subgroup analyses, when the length of the last follow-up was less than 3 years, there was no difference between two groups. However, as the last follow-up time increased, cervical Cobb angle was significantly lower in the ZP group when compared with the CP group.

**Conclusion:**

Based on the results of our analysis, the application of ZP device in ACDF had a lower rate of postoperative dysphagia and ASD than CP construct. Both devices were safe in anterior cervical surgeries, and they had similar efficacy in correcting radiologic outcomes. However, as the last follow-up time increased, ZP group showed greater changes cervical alignment. In order to clarify the specific significance of LOC, additional large clinical studies with longer follow-up period are required.

## Introduction

Anterior cervical diskectomy and fusion (ACDF) has been widely accepted as a gold standard for patients with obvious neurologic symptoms or ineffective conservative treatment, since the procedure was first described by Smith and Robinson [1]. Many studies described the advantages of ACDF as multifaceted, often involving a combination of direct decompression of neural structures, restoration of cervical sagittal alignment and continuously developing intervertebral instruments [2–6].

Traditionally, cage-plate (CP) construct is commonly used in ACDF. The use of polyetheretherketone (PEEK) cages for fusion has superseded bone graft because of the less complications associated with donor site infection, hematoma, and graft collapse [7]. The anterior plate is applied to enhance segmental stability, and restore cervical lordotic alignment [8]. Because cage-plate construct was proven to be efficient to treat cervical spondylotic myelopathy (CSM), it has been gradually applied in patients with multilevel CSM (MCSM) [9]. However, the treatment with a longer plate in clinical practice needs wider surgical exposure and more soft tissue injury, which are associated with functional impairments and comorbid conditions, such as trachea-esophageal injury, postoperative dysphagia, screw loosening or breakage, plate dislodgement, and adjacent segment degeneration(ASD) [10]. To avoid these plate-related complications, Zero-profile anchored spacer (ZP), consisting of a PEEK cage and 4 integrated screws, has been designed [11]. The design of no-plate implant allows the whole system into the intervertebral space and presents other advantages such as simplified operation and reduced operation time [12, 13].

In previous retrospective studies [2–6, 10, 14–18], researchers agreed that ZP and CP groups had similar clinical outcomes, both in terms of remission of symptoms and improvement in radiological results. Considering the potential risk of complications, some high-quality systematic reviews and meta-analyses suggested that Zero-P had an advantage in reducing the incidence of dysphagia and ASD [2, 16–18].

However, a trend of loss in restored cervical alignment and disk height during long time follow-up is beginning to concern many researchers, and a great loss of correction (LOC) might be related to changes in the biomechanics of the whole spine, long-term complications, and revision surgeries [13, 19].

Some research works [10, 13] demonstrated that LOC in cervical lordotic angle was larger in Zero-p groups within 3-year follow-up, but data from He et al. [5]'s study showed there was no difference in LOC between two groups. The results of LOC varied greatly among previous research works. Sun et al. [13] suggested that different numbers of treated segments, bias of personal skill, and small number of cases might contribute to the controversy. However, few studies have synthesized these multifaceted factors [20]. A meta-analysis by Liu et al. [21] focused on the subsidence, representing a loss of correction in disk height, in patients who had one-to-four levels of operation. One hundred and twenty-five cases were involved including 65 cases of Zero-P and 60 cases of PCC. The Zero-P group had a significantly higher subsidence rate than the CP group. The study noted that both single- and multilevel patients should be considered, but did not efforts to assess other parameters of cervical sagittal alignment, which is necessary to inform future efforts.

In order to advance clinical evidence based on new data and address the limitations of the previous reviews, we conducted a meta-analysis focused on evaluating two major questions: (1) Whether the LOC in cervical sagittal alignment is different between two groups during follow-up? and (2) Whether the LOC influence the clinical results and relate to long-term complications? This article was the first meta-analysis comparing the changes of radiographic parameters in patients using ZP devices with CP fixation during follow-up. Our goal was to synthesize prior research works and provide evidence for clinicians to make clinical decisions.

## Methods

This meta-analysis was conducted under the guidelines of the Review Manager handbook from the Cochrane Collaboration and was performed in accordance with the PRISMA Statement.

### Search strategy

A systematic search of the literature was conducted in the Cochrane Library, Embase.com, MEDLINE, PubMed, Springer, and Web of Science. Electronic databases were searched for relevant reports published up to July 2022. Boolean operators were used as follows: ((("Spondylosis"[Mesh]) OR (((((((Lumbarsacral Spondylosis[Title/Abstract]) OR (Spondylosis, Lumbarsacral[Title/Abstract])) OR (Thoracic Spondylosis[Title/Abstract])) OR (Spondylosis, Thoracic[Title/Abstract])) OR (Cervical Spondylosis[Title/Abstract])) OR (Spondylosis, Cervical[Title/Abstract])) OR (Spondylosis Deformans[Title/Abstract]))) AND ((Anterior cervical discectomy and fusion[Title/Abstract]) OR ((ACDF[Title/Abstract]) OR (anterior cervical decompression and fusion[Title/Abstract])))) AND (((((((Zero- profile[Title/Abstract]) OR (Zero-p[Title/Abstract])) OR (Zero profile[Title/Abstract])) OR (Stand-alone[Title/Abstract])) OR (anchored spacer[Title/Abstract])) OR (anchored cage[Title/Abstract])) OR (no- profile[Title/Abstract])) with no restriction of publication year and language. Reference lists of all included studies were scanned to identify additional potentially relevant research works.

### Inclusion and exclusion criteria

Two reviewers independently screened the titles and abstracts of identified papers and full-text copies of all potentially relevant studies, including randomized controlled trials (RCTs) and retrospective or prospective studies. The inclusion criteria for this study were as follows: (1) all patients with CSM (cervical spondylotic myelopathy) undergoing ACDF involving 1 level or more levels; (2) a direct comparison between the ZP implant and CP implant with clinical and radiological outcomes; (3) a follow-up time of no less than 12 months; and 4) patients aged ≥ 18 years. The exclusion criteria were as follows: (1) animal experiments, biomechanical studies, case reports, review articles, letters, and meeting abstracts (full text was not available); (2) combined anterior and posterior surgery or had a history of other cervical surgery; (3) duplicate publications; and (4) studies that did not meet the inclusion criteria. Disagreements were resolved through discussion until a consensus was reached.

### Data extraction

Two reviewers independently extracted data from the studies included in accordance with the above requirements, using the following categories: (1) Basic characteristics: publication year, study design, enrolled number, gender, age, operative levels, and follow-up duration. (2) Clinical outcomes: dysphagia rate, other complication types, and complication rates. (3) Radiological results, radiological parameters were recorded before and after surgery and during follow-up for comparison, such as cervical Cobb angle, incidence of subsidence, intervertebral disk height, adjacent segment degeneration (ASD), and fusion rate.

### Quality assessment

Two authors assessed the methodological quality of each included study independently. Newcastle–Ottawa Scale was used to assess the quality of retrospective and prospective studies because of its evaluation of three items (selection, comparability, and exposure). This scale had a maximum of 9 points, Studies with a ≥ 6 score were considered to be of relatively high quality [8].

### Statistical analysis

Statistical analysis was conducted using the Review Manager software (RevMan Version 5.4.1; Cochrane Collaboration). For continuous data, mean difference (MD) and 95% confidence interval (CI) were assessed. Odds Ratios (ORs) and 95% confidence intervals (CIs) were used for the analysis of dichotomous outcomes. The level of significance was set at *P* < 0.05. Heterogeneity was evaluated using the χ2 test and I2 statistics (heterogeneity was considered to be detected when *P* < 0.10 or I2 > 50%). When the heterogeneity was significant, the random effect model was used. Funnel plots were performed to evaluate whether there was publication bias. The sensitivity analysis was employed to test the strength and robustness of pooled results.

### Subgroup analyses

We performed several subgroup analyses to test interactions according to the level of ACDF surgery (1 and ≥ 2 level). At the same time, we conducted retrospective subgroup analyses based on length of follow-up (< 3 years and ≥ 3 years) to compare the radiological changes of different surgical procedures in different rehabilitation time longitudinally.

## Results

### Literature results and study characteristics

**Fig. 1:**
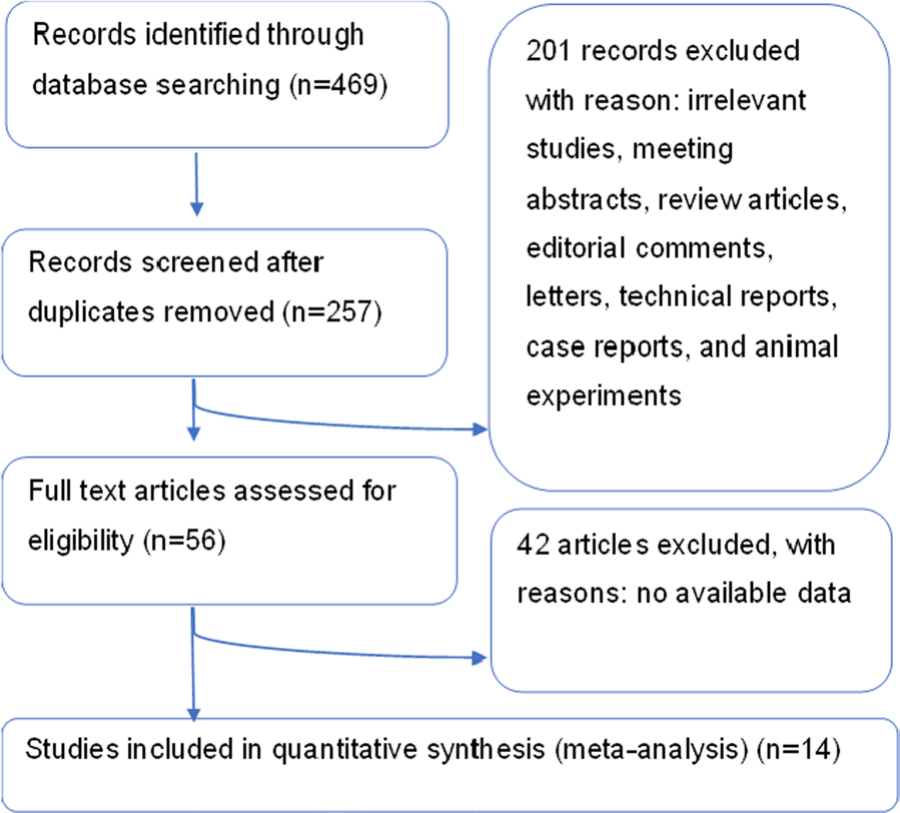
Search strategy and final included and excluded studies

A total of 432 studies were identified according to the search strategy initially, and included 14 eligible trials in the final meta-analysis (Fig. 1). All 14 studies are retrospective or prospective (retrospective studies, 11 studies; prospective studies, 3 studies). Table 1 shows the summary and basic characteristics of included studies. A total of 1067 patients (ZP group: 503 vs. CP group: 564) were evaluated. The surgical level ranged from 1 to 3, and all the lengths of follow-up were more than 12 months. There were no statistically significant differences for patient age and gender of all 14 studies. Our investigators evaluated each study with Newcastle–Ottawa Scale assessment. All studies scored ≥ 7 points (Table 1), which indicated that all the studies were of a relatively high quality.

**Table 1:** Characteristics of the 14 studies included in this meta-analysis

References	Design	Number of patients		Surgical level	Average follow-up (mos)		Mean ages (years)		Quality assessment (NOQAS)			
		ZP	CP		ZP	CP	ZP	CP	Selection	Comparability	Exposure	Total score
Chen et al. [2]	P	37	32	2	40.6 ± 9.2	43.5 ± 10.4	48.9 ± 4.0	49.5 ± 4.2	3	2	3	8
Chen et al. [3]	P	34	38	3	36	36	56.2 ± 5.7	56.9 ± 5.9	4	2	3	9
Chen et al. [4]	R	33	38	3	30.0 ± 5	31.5 ± 4.5	49.3 ± 3.7	48.8 ± 3.9	3	2	3	8
He et al. [5]	P	52	52	2,3,4	24	24	55.4 ± 12.4	59.5 ± 12.6	3	1	3	7
Lan et al. [6]	R	35	33	1	23.68 ± 1.93	24.39 ± 2.00	54.05 ± 10.11	52.09 ± 10.46	4	1	3	8
Li et al. [14]	R	61	55	1	24	24	58.8 ± 4.6	58.5 ± 4.9	4	2	3	9
Liu et al. [15]	R	28	32	3,4	23.3 ± 6.9	24.2 ± 6.4	56.6 ± 9.7	57.5 ± 9.5	3	2	3	8
Lu et al. [10]	R	22	24	2	30.5 ± 5.2	32.1 ± 6.5	56.6 ± 6.4	58.6 ± 7.2	3	2	3	8
Qi et al. [11]	R	83	107	1,2,3	17.9 ± 4.2	19.0 ± 4.2	42.3	42.5	3	1	3	7
Shi et al. [12]	R	34	31	2	24	24	56.1 ± 4.5	55.8 ± 4.9	4	2	3	9
Shi et al. [13]	R	27	34	3	60	60	54.7 ± 7.6	56.4 ± 7.5	3	2	3	8
Wang et al. [17]	R	22	25	1	33.59 ± 5.52	33.16 ± 5.97	50.86 ± 8.79	53.68 ± 8.96	3	2	3	8
Wang et al. [16]	R	27	30	1	35.2	35.5	51.6 ± 11.3	54.0 ± 8.5	3	1	3	7
Zhang et al. [18]	R	74	116	1	34.07 ± 3.20	36.50 ± 6.28	50.14 ± 6.05	50.29 ± 9.06	3	2	3	8

P, prospective study; R, retrospective study

### IDH

**Fig. 2:**
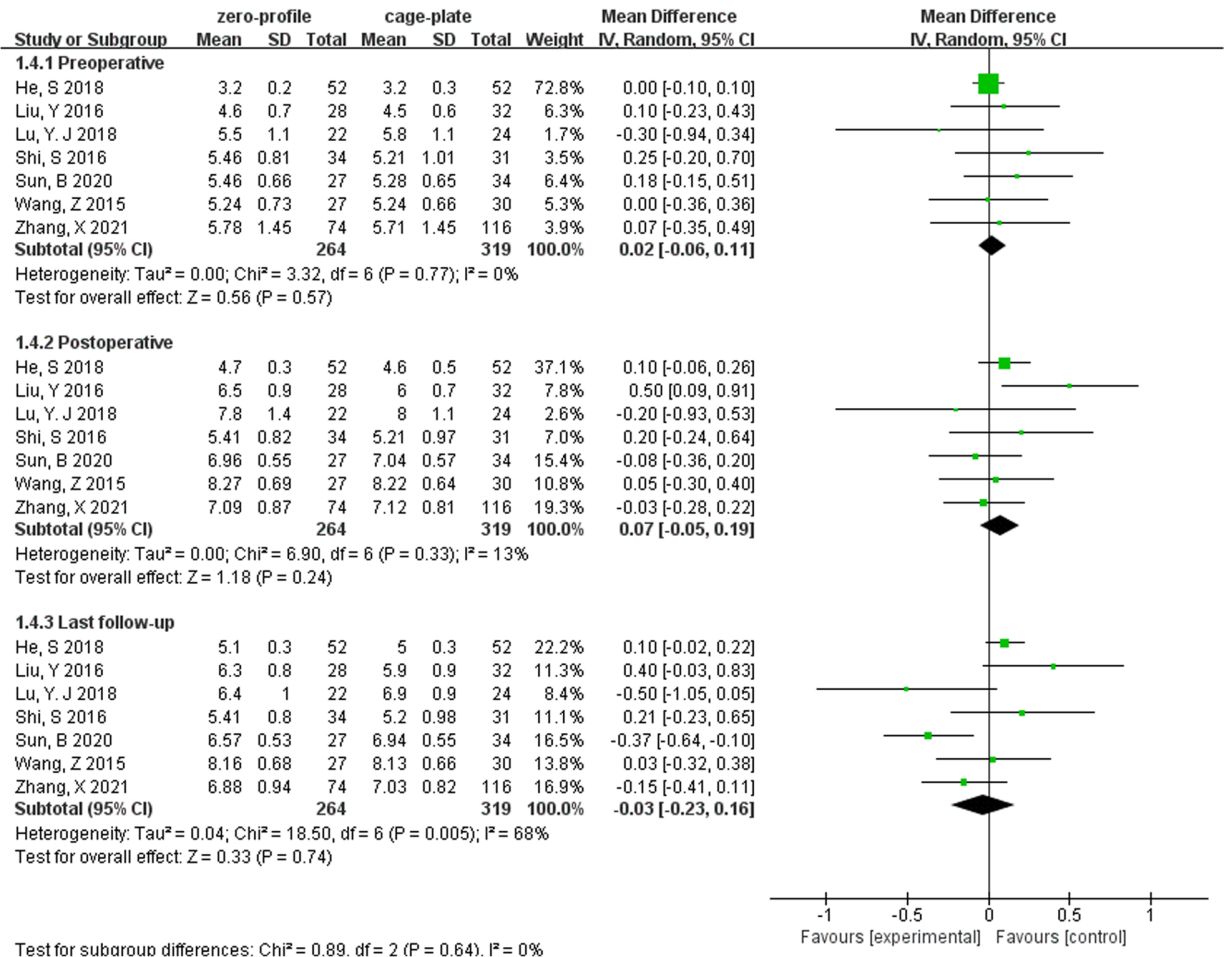
Forest plot of IDH between the ZP group and CP group

Seven studies consisting of 583 patients reported preoperative, postoperative, and last follow-up intervertebral disk height (ZP group, 264; CP group, 319). The random-effects model was applied and there was no significant difference in preoperative (MD, 0.02; 95% CI − 0.06 to 0.11; *P* = 0.57; *I*^2^ = 0%), postoperative (MD, 0.07; 95% CI − 0.05 to 0.19; *P* = 0.24; *I*^2^ = 13%) and last follow-up IDH (MD, 0.02; 95% CI − 0.03 to 0.23; *P* = 0.74; *I*^2^ = 68%) (Fig. 2).

### Cobb

**Fig. 3:**
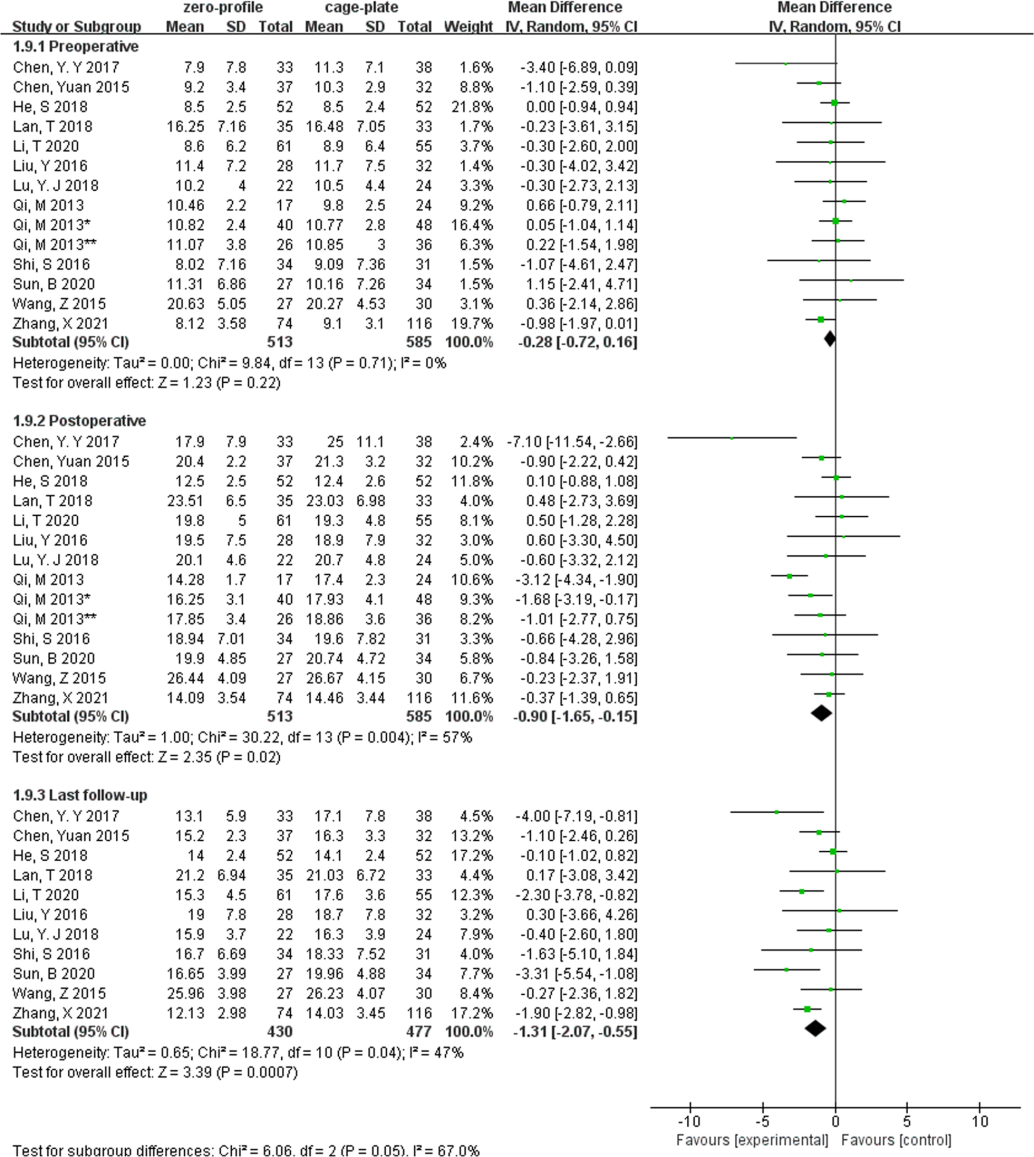
Forest plot of cervical Cobb angle between the ZP group and CP group

The changes in cervical Cobb angle were analyzed in 12 studies, which involved 1098 patients (ZP group, 513; CP group, 585). There was no difference in cervical Cobb angle between the ZP group and CP group before surgery without heterogeneity (MD, − 0.28; 95% CI − 0.72 to 0.16; *P* = 0.22; *I*^2^ = 0%). However, postoperative (MD, − 0.90; 95% CI − 1.65 to − 0.15; *P* = 0.02; *I*^2^ = 57%) and last follow-up cervical Cobb angle (MD, − 1.31; 95% CI − 2.07 to − 0.55; *P* = 0.0007; *I*^2^ = 47%) was significantly smaller in the ZP group when compared with the CP group (Fig. 3). Due to the pooled data from the relevant studies that showed evidence of median heterogeneity after surgery, the random-effects model was applied.

### ASD

**Fig. 4:**
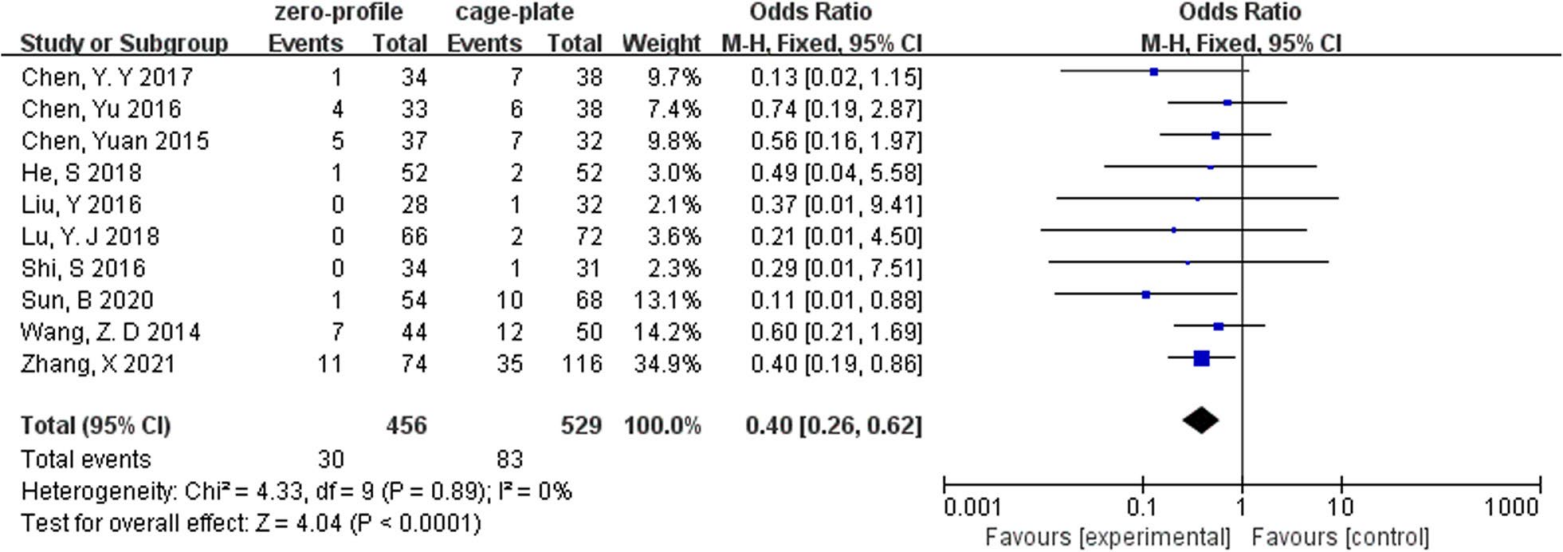
Forest plot of ASD between the ZP group and CP group

The incidence of adjacent segment degeneration was analyzed in 10 studies, which involved 985 patients (ZP group, 456; CP group, 529). The fixed-effects model was applied and the comparison revealed that the incidence of ASD was significantly lower in the ZP group with no evidence of statistical heterogeneity (OR, 0.40; 95% CI 0.26 to 0.62; *P* < 0.0001; *I*^2^ = 0%) (Fig. 4).

### Dysphagia

**Fig. 5:**
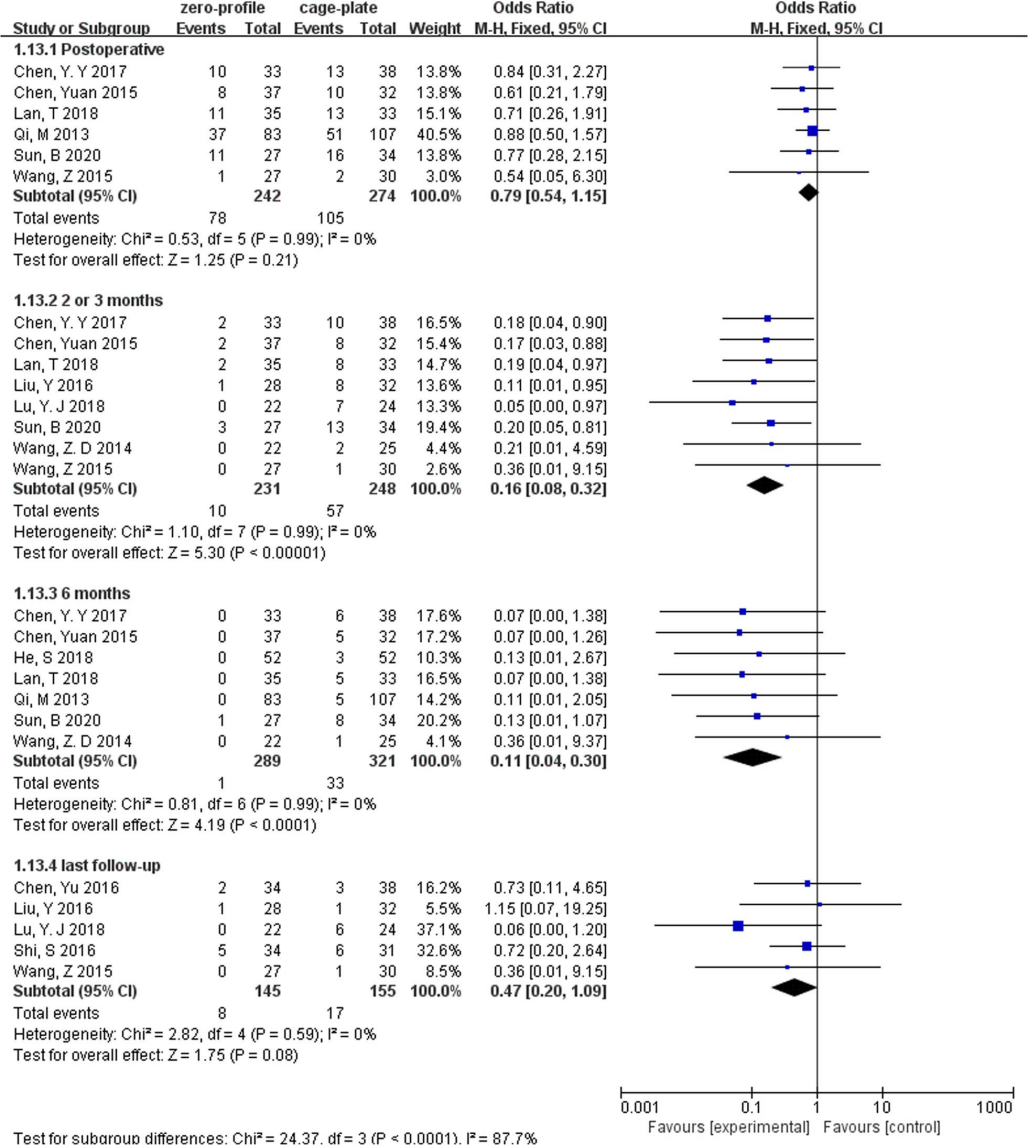
Forest plot of postoperative dysphagia between the ZP group and CP group

A total of 11 studies reported postoperative dysphagia during follow-up. ZP group and CP group were compared on the first 2 days after surgery (ZP group, 242; CP group, 274), the 2- or 3-month follow-up (ZP group, 231; CP group, 248), the 6-month follow-up (ZP group, 289; CP group, 321) and the last follow-up (ZP group, 145; CP group, 155). Due to the pooled data from the relevant studies that showed no evidence of statistical heterogeneity during follow-up (*I*^2^ = 0%), the fixed-effects model was applied. The ZP group was not associated with a significantly different likelihood of postoperative dysphagia at the first 2 days after surgery (OR, 0.79; 95% CI 0.54–1.15; *P* = 0.21) and at the last follow-up (OR, 0.47; 95% CI 0.20–1.09; *P* = 0.08) when compared with CP group. However, a significantly low rate of dysphagia was found in ZP group at the 2- or 3-month follow-up (OR, 0.16; 95% CI 0.08–0.32; *P* < 0.00001) and at the 6-month follow-up (OR, 0.11; 95% CI 0.04–0.30; *P* < 0.00001) (Fig. 5).

### Subgroup analyses

**Fig. 6:**
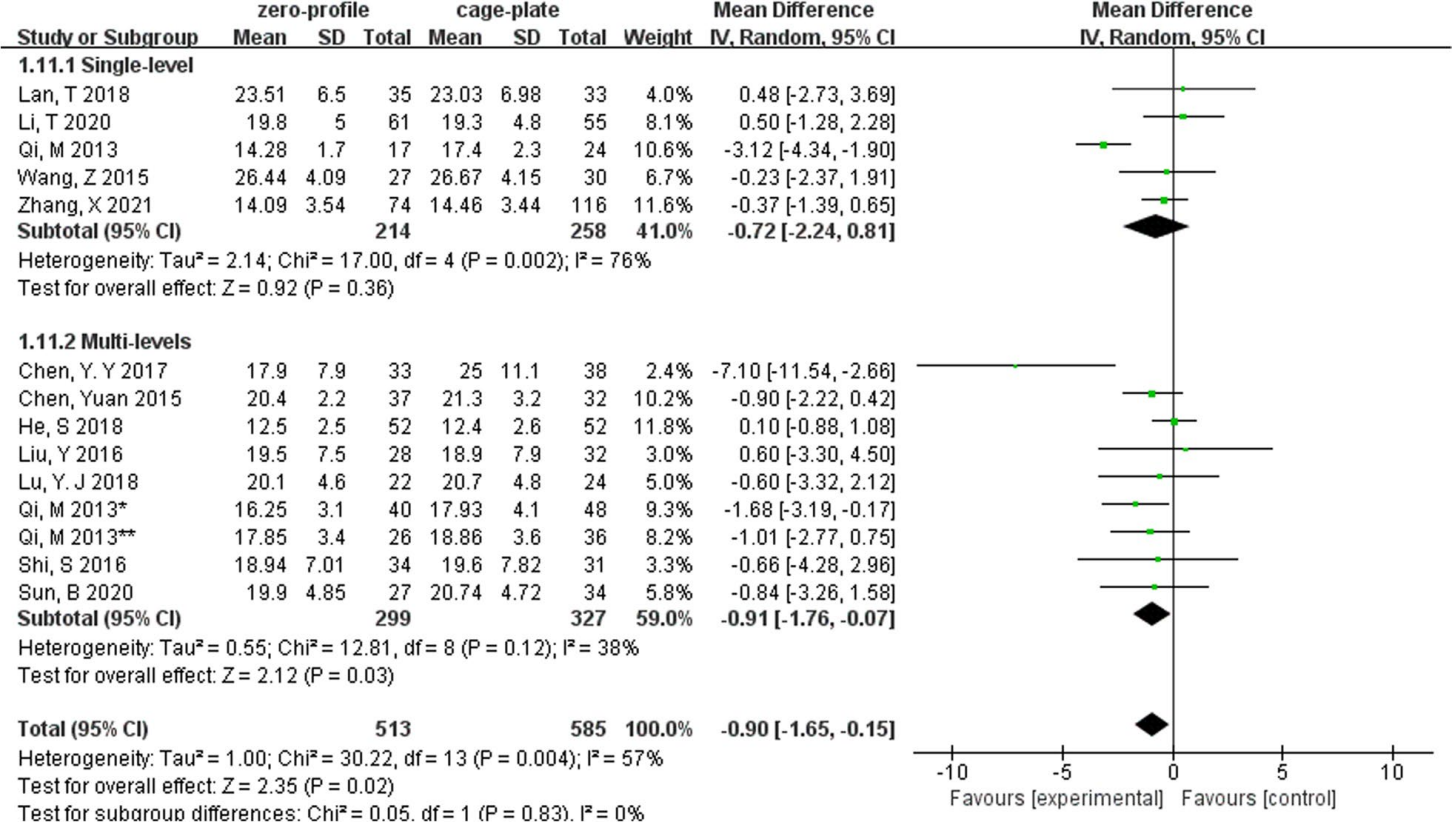
Forest plot of subgroup analyses of postoperative cervical Cobb angle according to levels of ACDF surgery

**Fig. 7:**
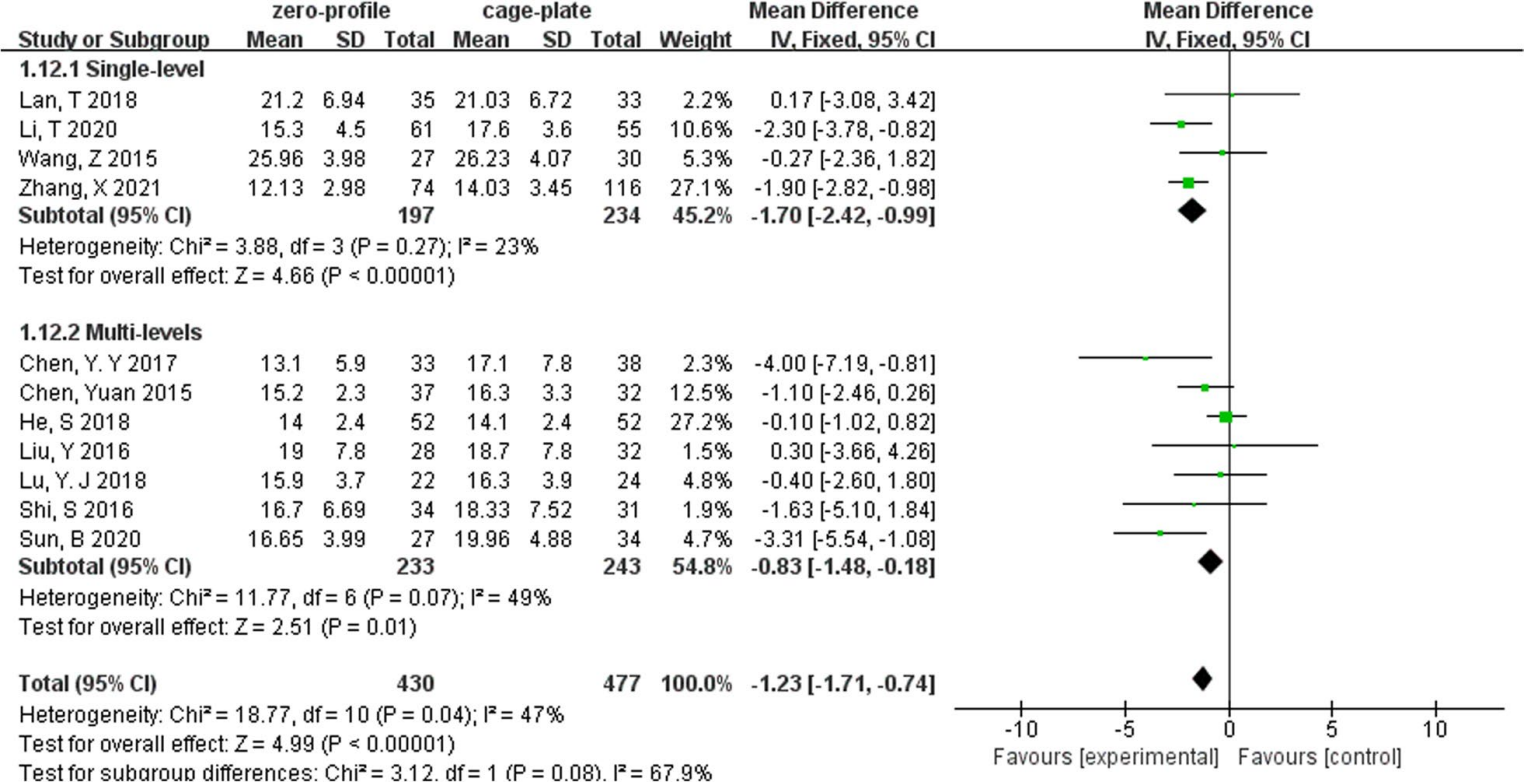
Forest plot of subgroup analyses of the last follow-up cervical Cobb angle according to levels of ACDF surgery

**Fig. 8:**
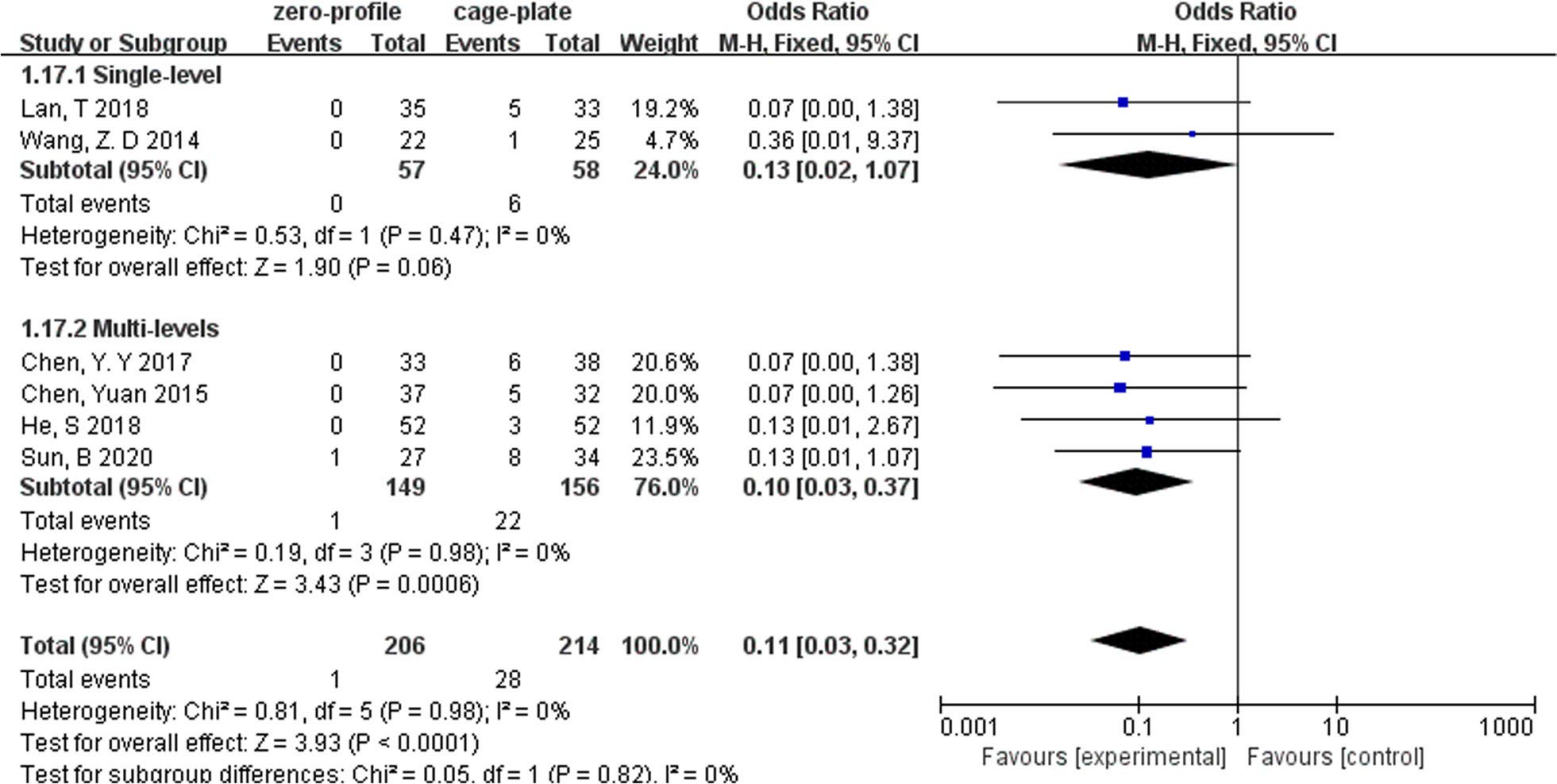
Forest plot of subgroup analyses of 6-month follow-up dysphagia according to levels of ACDF surgery

We performed subgroup analyses according to levels of ACDF surgery (Table 2). We found that in multilevel ACDF, postoperative cervical Cobb angle was significantly lower in the ZP group when compared with the CP group (MD, − 0.91; 95% CI − 1.76 to − 0.07; *P* = 0.03), although there was no difference between two groups in single-level ACDF (MD, − 0.72; 95% CI − 2.24 to 0.81; *P* = 0.36) (Fig. 6). In analyzing cervical Cobb Angle at the last follow-up, the subgroup heterogeneity played a role in contributing to overall heterogeneity (*I*^2^ = 67.9%) (Fig. 7). Different outcomes in subgroup analyses due to level of ACDF surgery were also seen in 6-month follow-up dysphagia (Fig. 8), there was no difference between two groups with single-level ACDF (OR, 0.13; 95% CI 0.02 to 1.07; *P* = 0.06). However, similar difference outcome in subgroup analyses was not reflected at the other follow-up time points. In subgroup analyses for dysphagia at the first 2 days after surgery and at the last follow-up, the ZP group was not associated with significantly different likelihood of postoperative dysphagia when compared with CP group regardless of the numbers of surgical level (Table 2). And a significantly low rate of dysphagia was found in ZP group at the 2- or 3-month follow-up regardless of the numbers of surgical level (Table 2).

**Table 2:** Subgroup analysis of the effect of different devices on cervical alignment

	Subgroup title	No. of studies	No. of participants (ZP)	No. of participants (CP)	Subgroup outcome			Test for subgroup differences: I^2^ (%)
					Subtotal (95%CI)	*P*	*I*^2^ (%)	
Cobb (preoperative)	No. of levels							
	1	5	214	258	− 0.36 [− 1.08, 0.36]	0.32	0	0
	≥ 2	8	299	327	− 0.23 [− 0.78, 0.33]	0.43	0	
Cobb (postoperative)	No. of levels							
	1	5	214	258	− 0.72 [− 2.24, 0.81]	0.36	76	0
	≥ 2	8	299	327	− 0.91 [− 1.76, − 0.07]	0.03*	38	
Cobb (last follow-up)	No. of levels							
	1	4	197	234	− 1.70 [− 2.42, − 0.99]	< 0.00001*	23	67.9
	≥ 2	7	233	243	− 0.83 [− 1.48, − 0.18]	0.01*	49	
	Years of the last follow-up							
	< 3	7	265	265	− 1.07 [− 2.21, 0.06]	0.06	46	0
	≥ 3	4	165	212	− 1.62 [− 2.56, − 0.67]	0.0008*	36	
IDH (preoperative)	No. of levels							
	1	2	101	146	0.03 [− 0.25, 0.30]	0.83	0	0
	≥ 2	5	163	173	0.02 [− 0.06, 0.11]	0.6	0	
IDH (postoperative)	No. of levels							
	1	2	101	146	− 0.00 [− 0.20, 0.20]	0.98	0	0
	≥ 2	5	163	173	0.10 [− 0.02, 0.22]	0.11	34	
IDH (last follow-up)	No. of levels							
	1	2	101	146	− 0.09 [− 0.29, 0.12]	0.42	0	0
	≥ 2	5	163	173	− 0.02 [− 0.31, 0.27]	0.89	76	
	Years of the last follow-up							
	< 3	4	136	139	0.09 [− 0.17, 0.36]	0.49	55	60.1
	≥ 3	3	128	180	− 0.18 [− 0.40, 0.03]	0.1	39	
ASD	No. of levels							
	1	3	170	218	0.54 [0.33, 0.88]	0.01*	0	0
	≥ 2	7	286	311	0.37 [0.20, 0.70]	0.002*	0	
	Years of the last follow-up							
	< 3	6	258	275	0.39 [0.18, 0.83]	0.01*	0	0
	≥ 3	4	198	254	0.41 [0.23, 0.70]	0.001*	0	
Dysphagia (postoperative)	No. of levels							
	1	2	62	63	0.68 [0.27, 1.71]	0.41	0	0
	≥ 2	3	97	104	0.74 [0.41, 1.34]	0.32	0	
Dysphagia (2 or 3 months)	No. of levels							
	1	3	84	88	0.21 [0.06, 0.80]	0.02*	0	0
	≥ 2	5	147	160	0.15 [0.07, 0.33]	< 0.00001*	0	
Dysphagia (6 months)	No. of levels							
	1	2	57	58	0.13 [0.02, 1.07]	0.06	0	0
	≥ 2	4	149	156	0.10 [0.03, 0.37]	0.0006*	0	
Dysphagia (last follow-up)	No. of levels							
	1	1	27	30	0.36 [0.01, 9.15]	0.53	–	0
	≥ 2	4	118	125	0.48 [0.20, 1.15]	0.1	0	

*Statistically significant

**Fig. 9:**
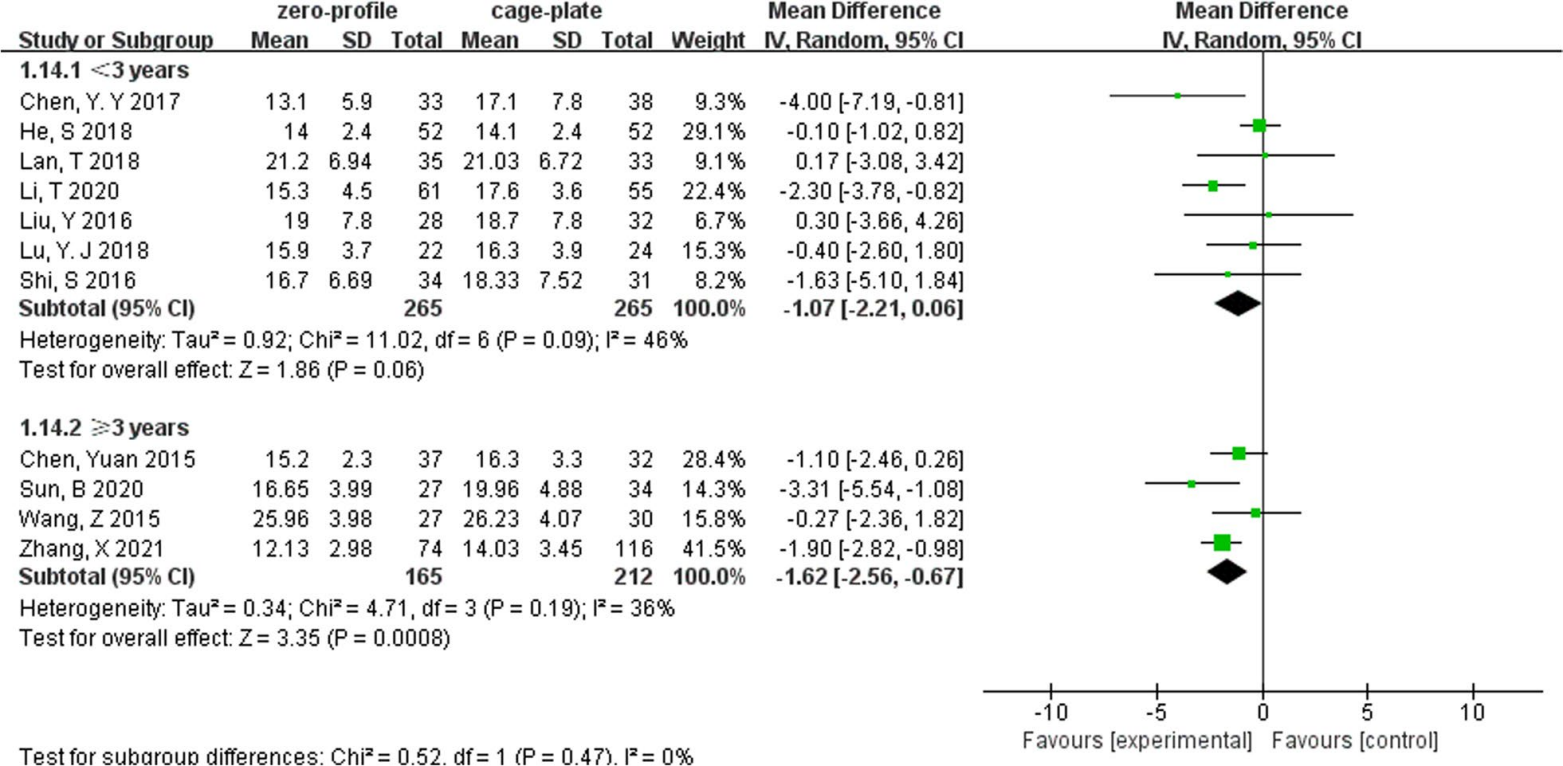
Forest plot of subgroup analyses of the last follow-up cervical Cobb angle according to the length of follow-up

We also conducted retrospective subgroup analyses based on the length of follow-up (Table 2). We found that when the length of the last follow-up was less than 3 years, there was no difference in cervical Cobb angle between two groups (MD, − 1.07; 95% CI − 2.21 to 0.06; *P* = 0.06). However, as the last follow-up time increased, the cervical Cobb angle was significantly lower in the ZP group when compared with the CP group (MD, − 1.62; 95% CI − 2.56 to − 0.67; *P* = 0.0008) (Fig. 9). In addition, the subgroup heterogeneity (*I*^2^ = 60.1%) contributed to overall heterogeneity of the last follow-up IDH (Table 2).

## Discussion

ACDF has been considered the standard operative treatment for cervical spondylotic myelopathy after the failure of conservative management, because it can provide direct anterior decompression and immediate stability to restore nerve function and rebuild cervical curvature [10, 11, 16–18, 22]. After being approved by the Food and Drug Administration (FDA) in 2008, Zero-P has been widely used in single-segment patients and gradually expanded to multi-segment patients [23]. In clinical practice, many clinicians believe that ZP has advantages in operation time, blood loss, length of hospital stay, and other aspects [5], which are reflected in the data of relevant retrospective studies [2–5]. A meta-analysis from Zhang et al. [8], synthesizing the literature on multilevel ACDF, demonstrates these advantages. Zhang et al. mentioned the difference may be related to the smaller surgical exposure and simpler operative procedures that multilevel ZP surgery required. In Shao et al.'s meta-analysis of single-segment ACDF [1], although there was no significant difference between ZP and CP in operation time, which indicated that the simplicity of ZP surgery was not clearly reflected in single-segment patients, he still recognized that ZP had a statistical significance reducing blood loss. Compared with cage-plate, ZP is advantageous because of the potential for less soft tissue contact in Shao's perspective [1]. And the less impact on prevertebral soft tissues such as esophagus can significantly reduce the risk of postoperative dysphagia, suggesting another advantage of ZP [24]. We abundantly overview the related work to demonstrate the difference in the incidence of dysphagia between the ZP and CP groups at four time points after operation, we found a significantly low rate of dysphagia in ZP group in our meta-analysis during the follow-up within 6 months. Our results suggest that the CP device may increase the incidence of dysphagia compared with the ZP device. Besides, the same studies comparing dysphagia results of the two devices in consecutive follow-up can also be interpreted as a longer dysphagia recovery time in patients with CP devices.

In addition to the advantages mentioned above, ZP has similar results to CP in the therapeutic efficacy and the encouraging clinical outcomes were reported by many researchers [2–6, 10–18]. Most patients in the ZP and CP groups received pain relief and neurological function recovery postoperatively, which was reflected in the similar improvement in JOA score and NDI score in both groups between the pre- and immediate postoperative time points [2, 10, 13]. Meanwhile, at the short-time follow-up of the two groups in our research, radiological parameters representing the cervical sagittal alignment, such as IDH and Cobb angle, were also significantly improved, in both single- and multilevel studies. From the perspectives of clinical and radiological results, we demonstrated that ZP and CP have similar and excellent effectiveness. Most of what we know about the complications associated with surgical procedures, such as hematoma, screw loosening, dislocation, infection, C5 palsy, hoarseness, epidural hematoma, and cerebral fluid leakage, remain rarely reported. It might indicate that both ZP and CP surgeries are relatively safe. Based on the above views, we believe that ZP shows similar efficacy and safety to CP during short-term follow-up.

However, as the length of follow-up time has increased, some researchers have raised concerns about the increasing use of ZP devices [7, 13, 25]. Plenty of previous studies on the discussion of safety and efficacy were based on results compared between pre- and postoperative time points, leading to the evidence gap in long-term changes of cervical alignment [13]. In a retrospective study with long-term follow-up by Sun's team [13], they mentioned that disc height and Cobb angle were well restored after operations, but lost in both ZP and CP groups during follow-up. Combining previous research works, they noticed the prevalence of the phenomenon and decided to quantify the loss of correction (LOC). In terms of quantitative measurement, LOC of cervical lordosis in ZP group constantly grew from 11.28 to 48.13% during the 5-year follow-up, and as for qualitative comparison, LOC in ZP group was significantly higher than that in the CP group. Some other researchers conducted retrospective studies on single- and multilevel patients, respectively, and also demonstrated that the loss in cervical lordosis was larger with ZP [4, 13, 14, 18]. However, after integrating the four studies, Liu et al. [21] reported that there was no significant difference in CL between the two groups in the 12th month. In terms of LOC, the sample sizes of the above retrospective study and meta-analysis were relatively small, and the length of follow-up time was of great heterogeneity [8, 21]. The inconclusive debate still remains. Therefore, synthesizing high-quality studies with medium—and long-term follow-up are important to fill critical knowledge gaps in the change of cervical alignment and can evaluate the long-term efficacy of the two devices from a relatively comprehensive perspective that may provide evidence for clinicians to make clinical decisions. We conducted retrospective subgroup analyses based on the length of follow-up (< 3 and ≥ 3 years of the last follow-up). We found that when the length of the last follow-up was less than 3 years, there was no difference between two groups. However, as the last follow-up time increased, the cervical Cobb angle was significantly lower among studies with Zero-p than in studies with CP. We verified the existence of LOC through meta-analysis. In addition, and with the increase in follow-up time, the difference between the two groups was more obvious. Although the exact pathophysiologic mechanism of LOC remains unclear, according to Kinon et al. [26], plate could combine all vertebrae and instruments work as a whole, by working as a frame resisting axial compression, especially with screws on multilevel vertebrae. Meanwhile, biomechanical research works found that the plate could provide better segmental stability than Zero-profile spacer [27, 28], which was consistent with our findings.

Loss of CL is often considered to cause progressive degenerative cervical spondylosis (DCS) and also be responsible for neck pain and neurological dysfunction [5, 29–32]. However, the higher LOC after ACDF with ZP device, especially the data of LOC in CL as high as 48.13% given by Sun et al. [13] in the 5-year follow-up, makes us worry about the condition of patients in the longer term. Although we have collected a large number of relevant studies, our findings still starkly amplify the paucity of long-term follow-up evidence that provides important insights on the late-term effects of LOC, and effective strategies to address these problems remain woefully underdeveloped. Instead of the existing approach to retrospective study, we offer the following underutilized solutions to expand clinical trials to analyze the changes in cervical sagittal alignment. (1) Leverage prospective cohort studies and multicenter studies to increase sample sizes and ensure the adequate length of follow-up. (2) Quantifying the changes in radiological results and increasing the use of scales to evaluate clinical outcomes during long-term follow-up. (3) Record the late-term complications and revision surgeries and analyze the causes and relationship to the LOC individually. (4) Increase the use of finite element models to assist the analysis of the changes in sagittal alignment [33, 34].

Our study has limitations. First, among enrolled studies, there were few randomized controlled trials and prospective cohort studies. Therefore, more studies with large sample size, long-term follow-up, and high quality are still needed to confirm the results. Second, statistical heterogeneity was detected and it might be explained by the study design, the patients' characteristics, surgical techniques, and the length of follow-up. Lastly, all the included studies are in English language; thus, a potential language bias may exist in this meta-analysis.

Despite these limitations, our meta-analysis also has several strengths. First, to our knowledge, this is the first meta-analysis to synthesize the literature on the comparison of the changes in sagittal alignment between ZP and CP. Second, we specifically focus on the loss of correction during follow-up, which does exist and differs between groups, but is often neglected because of leading to few clinical symptoms. Meanwhile, we expand previous knowledge and provide new evidence for clinicians to make clinical decisions. Finally, we highlight several findings and recommendations aimed at directing future research and resources.

## Conclusion

Based on the results of our analysis, the application of the ZP device in ACDF had a lower rate of postoperative dysphagia and ASD than the CP construct. Both devices were safe in anterior cervical surgeries, and they had similar efficacy in correcting radiologic outcomes. However, as the last follow-up time increased, ZP group showed greater changes in cervical alignment. In order to clarify the specific significance of LOC, additional large clinical studies with longer follow-up period are required.

## Data Availability

All the data are included in the manuscript, and further data can be requested from the corresponding author upon reasonable request.
